# Dicyclo­hex­yl[4-(dimethyl­amino)­phen­yl]phosphine selenide

**DOI:** 10.1107/S1600536811054006

**Published:** 2011-12-23

**Authors:** Zanele H Phasha, Sizwe Makhoba, Alfred Muller

**Affiliations:** a Research Center for Synthesis and Catalysis, Department of Chemistry, University of Johannesburg (APK Campus), PO Box 524, Auckland Park, Johannesburg 2006, South Africa

## Abstract

In the title mol­ecule, C_20_H_32_NPSe, the P atom has a distorted tetra­hedral environment resulting in an effective cone angle of 172°. Weak inter­molecular C—H⋯Se inter­actions are observed.

## Related literature

For background to our investigation of the steric and electronic effects of group 15 ligands, see: Roodt *et al.* (2003 ▶); Muller *et al.* (2006 ▶, 2008 ▶). For background on cone angles, see: Bunten *et al.* (2002 ▶); Tolman (1977 ▶); Otto (2001 ▶).
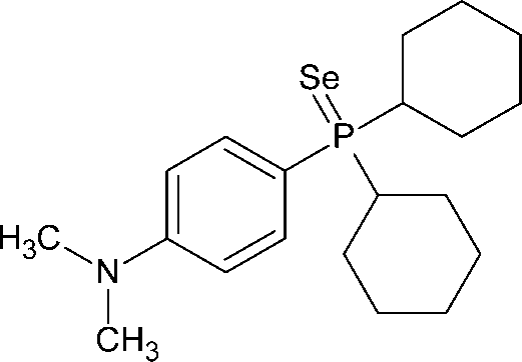


## Experimental

### 

#### Crystal data

C_20_H_32_NPSe
*M* *_r_* = 396.4
Monoclinic, 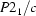

*a* = 12.3860 (16) Å
*b* = 6.8331 (8) Å
*c* = 24.113 (3) Å
β = 97.050 (3)°
*V* = 2025.3 (4) Å^3^
*Z* = 4
Mo *K*α radiation
μ = 1.93 mm^−1^
*T* = 100 K
0.29 × 0.12 × 0.05 mm

#### Data collection

Bruker APEX DUO 4K CCD diffractometer
Absorption correction: multi-scan (*SADABS*; Bruker, 2008 ▶) *T*_min_ = 0.604, *T*_max_ = 0.910
31168 measured reflections
5008 independent reflections
4192 reflections with *I* > 2σ(*I*)
*R*_int_ = 0.033

#### Refinement

*R*[*F* ^2^ > 2σ(*F* ^2^)] = 0.024
*wR*(*F* ^2^) = 0.063
*S* = 1.02
5008 reflections
210 parameters
H-atom parameters constrained
Δρ_max_ = 0.53 e Å^−3^
Δρ_min_ = −0.24 e Å^−3^

### 

Data collection: *APEX2* (Bruker, 2011 ▶); cell refinement: *SAINT* (Bruker, 2008 ▶); data reduction: *SAINT* and *XPREP* (Bruker, 2008 ▶); program(s) used to solve structure: *SIR97* (Altomare *et al.*, 1999 ▶); program(s) used to refine structure: *SHELXL97* (Sheldrick, 2008 ▶); molecular graphics: *DIAMOND* (Brandenburg & Putz, 2005 ▶); software used to prepare material for publication: *WinGX* (Farrugia, 1999 ▶).

## Supplementary Material

Crystal structure: contains datablock(s) global, I. DOI: 10.1107/S1600536811054006/nc2259sup1.cif

Structure factors: contains datablock(s) I. DOI: 10.1107/S1600536811054006/nc2259Isup2.hkl

Supplementary material file. DOI: 10.1107/S1600536811054006/nc2259Isup3.cml

Additional supplementary materials:  crystallographic information; 3D view; checkCIF report

## Figures and Tables

**Table 1:** Hydrogen-bond geometry (Å, °)

*D*—H⋯*A*	*D*—H	H⋯*A*	*D*⋯*A*	*D*—H⋯*A*
C15—H15⋯Se1^i^	1.00	2.71	3.6546 (15)	157
C19—H19*A*⋯Se1^i^	0.99	3.04	3.8836 (18)	143

Symmetry code: (i) 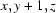.

**Fig. 1.**
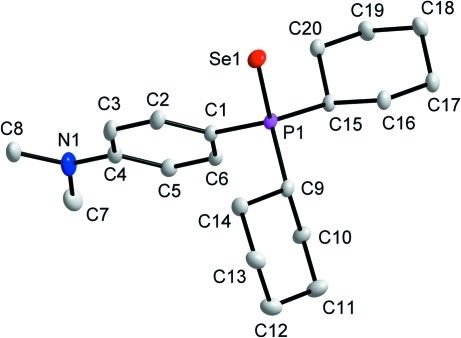
: View of the title compound with labelling and displacement ellipsoids drawn at a 50% probability level.

## supplementary crystallographic information

### Comment

The study of the transition metal phosphorous bond spans over several decades using various techniques such as crystallography, multi nuclear NMR and IR (Roodt *et al.*, 2003). As part of this systematic investigation we have extended this study to selenium derivatives of the phosphorus ligands (see Muller *et al.*, 2008). This way there is no steric crowding effect, albeit crystal packing effects, as normally found in transition metal complexes with bulky ligands, *e.g.* in *trans*-[Rh(CO)Cl{P(OC_6_H_5_)_3_}_2_] cone angles variation from 156° to 167° was observed for the two phosphite ligands (Muller *et al.*, 2006). The ^1^*J*(^31^P-^77^Se) coupling can also be used as an additional probe to obtain more information regarding the nature of the phosphorous bond. Reported as part of the above continuing study, the single-crystal structure of the phosphorus containing compound, SePCy_2_(4-N{CH_3_}_2_-C_6_H_4_) where Cy = C_6_H_11_, is reported here.

Molecules of the title compound (see Fig. 1) adopts a distorted tetrahedral arrangement about phosphorous atom with average C—P—C and Se—P—C angles of 107.1° and 111.9° respectively. Describing the steric demand of phosphane ligands has been the topic of many studies and a variety of models have been developed (Bunten *et al.*, 2002). The Tolman cone angle (Tolman, 1977) is still the most commonly used model. Applying this model to the geometry obtained for the title compound (and adjusting the Se—P bond distance to 2.28 Å) we calculated an effective cone angle (Otto, 2001) of 171.5°. Weak intermolecular C—H···Se interactions (Table 1) are observed in the crystal lattice.

### Experimental

Dicyclohexyl(4-(*N*,*N*-dimethylamino)phenyl)phosphine and KSeCN were purchased from Sigma-Aldrich and used without purification. Eqimolar amounts of KSeCN (5.8 mg, 0.04 mmol) and the dicyclohexyl(4-(*N*,*N*-dimethylamino)phenyl)phosphine (11.5 mg, 0.04 mmol) were dissolved in the minimum amounts of methanol (10 ml). The KSeCN solution was added drop wise (5 min.) to the phosphine solution with stirring at room temperature. The final solution was left to evaporate slowly until dry to give crystals suitable for a single-crystal X-ray study.

### Refinement

All hydrogen atoms were positioned in geometrically idealized positions with C—H = 1.00 Å (methine), 0.99 Å (methylene), 0.98 Å (methyl) and 0.95 Å (aromatic). All hydrogen atoms were allowed to ride on their parent atoms with *U*_iso_(H) = 1.2*U*_eq_, except for the methyl where *U*_iso_(H) = 1.5*U*_eq_ was utilized. The initial positions of methyl hydrogen atoms were located from a Fourier difference map and refined as fixed rotor. A solvent accesible void of 62 Å^3^ was detected by the checkcif routine. The residual electron density at this position is quite small and several attemps to refine a solvent molecule at this position failed, and was therefore left empty.

### Crystal data

**Table:**

C_20_H_32_NPSe	*F*(000) = 832
*M**_r_* = 396.4	*D*_x_ = 1.3 Mg m^−^^3^
Monoclinic, *P*2_1_/*c*	Mo *K*α radiation, λ = 0.71073 Å
Hall symbol: -P 2ybc	Cell parameters from 9647 reflections
*a* = 12.3860 (16) Å	θ = 2.2–28.2°
*b* = 6.8331 (8) Å	µ = 1.93 mm^−^^1^
*c* = 24.113 (3) Å	*T* = 100 K
β = 97.050 (3)°	Plate, colourless
*V* = 2025.3 (4) Å^3^	0.29 × 0.12 × 0.05 mm
*Z* = 4	

### Data collection

**Table:**

Bruker APEX DUO 4K CCD diffractometer	5008 independent reflections
graphite	4192 reflections with *I* > 2σ(*I*)
Detector resolution: 8.4 pixels mm^-1^	*R*_int_ = 0.033
φ and ω scans	θ_max_ = 28.3°, θ_min_ = 1.7°
Absorption correction: multi-scan (*SADABS*; Bruker, 2008)	*h* = −16→16
*T*_min_ = 0.604, *T*_max_ = 0.910	*k* = −9→7
31168 measured reflections	*l* = −32→32

### Refinement

**Table:**

Refinement on *F*^2^	Primary atom site location: structure-invariant direct methods
Least-squares matrix: full	Secondary atom site location: difference Fourier map
*R*[*F*^2^ > 2σ(*F*^2^)] = 0.024	Hydrogen site location: inferred from neighbouring sites
*wR*(*F*^2^) = 0.063	H-atom parameters constrained
*S* = 1.02	*w* = 1/[σ^2^(*F*_o_^2^) + (0.0282*P*)^2^ + 1.2459*P*] where *P* = (*F*_o_^2^ + 2*F*_c_^2^)/3
5008 reflections	(Δ/σ)_max_ = 0.001
210 parameters	Δρ_max_ = 0.53 e Å^−^^3^
0 restraints	Δρ_min_ = −0.24 e Å^−^^3^

### Special details

**Table:**

Experimental. The intensity data was collected on a Bruker Apex DUO 4 K CCD diffractometer using an exposure time of 30 s/frame. A total of 1671 frames were collected with a frame width of 0.5° covering up to θ = 28.29° with 99.8% completeness accomplished. Analytical data: ^1^H NMR (CDCl_3_, 400 MHz) δ 7.68, 6.69 (m, 4H), 2.99 (s, 6H), 2.19–1.13 (m, 22H); ^13^C {H} NMR (CDCl_3_, 100 MHz) δ 152.2, 134.2, 111.0 (Ar); 40.0 (Me); 36.72, 26.36 (Cy); ^31^P {H} NMR (CDCl_3_, 160 MHz):δ = 52.9 (t, ^1^*J*_Se—P_ = 687 Hz, 1P).
Geometry. All s.u.'s (except the s.u. in the dihedral angle between two l.s. planes) are estimated using the full covariance matrix. The cell s.u.'s are taken into account individually in the estimation of s.u.'s in distances, angles and torsion angles; correlations between s.u.'s in cell parameters are only used when they are defined by crystal symmetry. An approximate (isotropic) treatment of cell s.u.'s is used for estimating s.u.'s involving l.s. planes.
Refinement. Refinement of *F*^2^ against ALL reflections. The weighted *R*-factor *wR* and goodness of fit *S* are based on *F*^2^, conventional *R*-factors *R* are based on *F*, with *F* set to zero for negative *F*^2^. The threshold expression of *F*^2^ > 2σ(*F*^2^) is used only for calculating *R*-factors(gt) *etc*. and is not relevant to the choice of reflections for refinement. *R*-factors based on *F*^2^ are statistically about twice as large as those based on *F*, and *R*- factors based on ALL data will be even larger.

### Fractional atomic coordinates and isotropic or equivalent isotropic displacement parameters (Å^2^)

**Table:**

	*x*	*y*	*z*	*U*_iso_*/*U*_eq_	
Se1	0.198411 (13)	0.25268 (2)	0.085698 (7)	0.01880 (5)	
P1	0.17385 (3)	0.54119 (5)	0.114465 (16)	0.01278 (8)	
C2	0.30292 (12)	0.4535 (2)	0.21383 (6)	0.0180 (3)	
H2	0.2882	0.3209	0.2037	0.022*	
N1	0.45558 (12)	0.7312 (2)	0.32915 (6)	0.0252 (3)	
C1	0.26027 (12)	0.6017 (2)	0.17787 (6)	0.0139 (3)	
C3	0.36616 (13)	0.4943 (2)	0.26382 (7)	0.0198 (3)	
H3	0.3931	0.3898	0.2876	0.024*	
C4	0.39116 (12)	0.6884 (2)	0.28002 (6)	0.0179 (3)	
C5	0.34835 (12)	0.8385 (2)	0.24342 (6)	0.0175 (3)	
H5	0.3638	0.9713	0.2531	0.021*	
C6	0.28439 (12)	0.7956 (2)	0.19382 (6)	0.0149 (3)	
H6	0.2563	0.8994	0.1701	0.018*	
C7	0.46054 (15)	0.9295 (3)	0.35068 (7)	0.0279 (4)	
H7A	0.4903	1.0163	0.324	0.042*	
H7B	0.5074	0.9331	0.3865	0.042*	
H7C	0.3872	0.9732	0.3561	0.042*	
C8	0.49523 (14)	0.5753 (3)	0.36689 (7)	0.0269 (4)	
H8A	0.4342	0.5178	0.3835	0.04*	
H8B	0.5485	0.6281	0.3965	0.04*	
H8C	0.5298	0.4744	0.3462	0.04*	
C9	0.03310 (12)	0.5742 (2)	0.12921 (6)	0.0144 (3)	
H9	−0.0153	0.5443	0.0939	0.017*	
C10	0.00619 (13)	0.7830 (2)	0.14649 (7)	0.0189 (3)	
H10A	0.0529	0.8187	0.1813	0.023*	
H10B	0.0212	0.8761	0.1169	0.023*	
C11	−0.11370 (13)	0.7973 (3)	0.15585 (8)	0.0242 (4)	
H11A	−0.1286	0.9305	0.1692	0.029*	
H11B	−0.16	0.7763	0.1199	0.029*	
C12	−0.14340 (14)	0.6474 (3)	0.19833 (7)	0.0234 (3)	
H12A	−0.2224	0.6548	0.201	0.028*	
H12B	−0.1042	0.6788	0.2355	0.028*	
C13	−0.11435 (13)	0.4399 (2)	0.18195 (7)	0.0201 (3)	
H13A	−0.1602	0.4021	0.147	0.024*	
H13B	−0.1295	0.3475	0.2117	0.024*	
C14	0.00578 (12)	0.4261 (2)	0.17331 (6)	0.0178 (3)	
H14A	0.022	0.2921	0.1611	0.021*	
H14B	0.0517	0.4522	0.2091	0.021*	
C15	0.19907 (12)	0.7244 (2)	0.06209 (6)	0.0138 (3)	
H15	0.1885	0.8568	0.0783	0.017*	
C16	0.11808 (12)	0.7031 (2)	0.00891 (6)	0.0170 (3)	
H16A	0.1222	0.5685	−0.0059	0.02*	
H16B	0.0433	0.7244	0.0182	0.02*	
C17	0.14214 (13)	0.8499 (2)	−0.03583 (6)	0.0194 (3)	
H17A	0.0914	0.8273	−0.0703	0.023*	
H17B	0.1301	0.9844	−0.0226	0.023*	
C18	0.25890 (13)	0.8306 (3)	−0.04903 (7)	0.0236 (3)	
H18A	0.2694	0.6997	−0.0651	0.028*	
H18B	0.2729	0.9302	−0.0771	0.028*	
C19	0.33917 (13)	0.8580 (3)	0.00356 (7)	0.0211 (3)	
H19A	0.3329	0.9928	0.0179	0.025*	
H19B	0.4143	0.84	−0.0056	0.025*	
C20	0.31696 (12)	0.7111 (2)	0.04871 (6)	0.0172 (3)	
H20A	0.3673	0.7367	0.0831	0.021*	
H20B	0.3312	0.577	0.0359	0.021*	

### Atomic displacement parameters (Å^2^)

**Table:**

	*U*^11^	*U*^22^	*U*^33^	*U*^12^	*U*^13^	*U*^23^
Se1	0.02229 (8)	0.00922 (8)	0.02423 (9)	0.00091 (6)	0.00027 (6)	−0.00182 (6)
P1	0.01404 (17)	0.00898 (16)	0.01457 (17)	−0.00026 (14)	−0.00125 (13)	0.00036 (14)
C2	0.0184 (7)	0.0145 (7)	0.0201 (7)	−0.0009 (6)	−0.0014 (6)	0.0038 (6)
N1	0.0244 (7)	0.0300 (8)	0.0187 (7)	−0.0028 (6)	−0.0079 (5)	0.0012 (6)
C1	0.0128 (6)	0.0141 (7)	0.0143 (7)	−0.0005 (5)	−0.0009 (5)	0.0011 (6)
C3	0.0185 (7)	0.0208 (8)	0.0190 (7)	0.0004 (6)	−0.0020 (6)	0.0064 (6)
C4	0.0124 (7)	0.0253 (8)	0.0153 (7)	−0.0016 (6)	−0.0014 (5)	0.0014 (6)
C5	0.0168 (7)	0.0160 (7)	0.0191 (7)	−0.0021 (6)	0.0001 (6)	−0.0008 (6)
C6	0.0145 (7)	0.0143 (7)	0.0156 (7)	0.0005 (5)	0.0002 (5)	0.0021 (5)
C7	0.0258 (9)	0.0357 (10)	0.0203 (8)	−0.0039 (8)	−0.0047 (7)	−0.0057 (7)
C8	0.0219 (8)	0.0409 (11)	0.0166 (8)	0.0064 (8)	−0.0028 (6)	0.0033 (7)
C9	0.0143 (7)	0.0131 (7)	0.0147 (7)	−0.0011 (5)	−0.0029 (5)	0.0007 (5)
C10	0.0173 (7)	0.0141 (7)	0.0251 (8)	0.0014 (6)	0.0020 (6)	0.0008 (6)
C11	0.0189 (8)	0.0210 (8)	0.0332 (9)	0.0038 (6)	0.0048 (7)	−0.0009 (7)
C12	0.0219 (8)	0.0242 (9)	0.0248 (8)	−0.0023 (7)	0.0054 (6)	−0.0063 (7)
C13	0.0213 (8)	0.0198 (8)	0.0197 (7)	−0.0053 (6)	0.0048 (6)	−0.0033 (6)
C14	0.0198 (7)	0.0145 (7)	0.0188 (7)	−0.0022 (6)	0.0014 (6)	0.0013 (6)
C15	0.0168 (7)	0.0103 (7)	0.0133 (6)	−0.0007 (5)	−0.0016 (5)	0.0009 (5)
C16	0.0168 (7)	0.0174 (7)	0.0154 (7)	−0.0020 (6)	−0.0038 (6)	0.0011 (6)
C17	0.0215 (8)	0.0199 (8)	0.0157 (7)	0.0001 (6)	−0.0026 (6)	0.0024 (6)
C18	0.0243 (8)	0.0317 (9)	0.0145 (7)	0.0002 (7)	0.0019 (6)	0.0036 (7)
C19	0.0189 (7)	0.0260 (9)	0.0184 (7)	−0.0035 (6)	0.0019 (6)	0.0031 (7)
C20	0.0155 (7)	0.0194 (8)	0.0159 (7)	−0.0006 (6)	−0.0015 (5)	0.0007 (6)

### Geometric parameters (Å, °)

**Table:**

Se1—P1	2.1241 (5)	C11—C12	1.525 (2)
P1—C1	1.8034 (15)	C11—H11A	0.99
P1—C15	1.8321 (15)	C11—H11B	0.99
P1—C9	1.8354 (15)	C12—C13	1.527 (2)
C2—C3	1.383 (2)	C12—H12A	0.99
C2—C1	1.394 (2)	C12—H12B	0.99
C2—H2	0.95	C13—C14	1.531 (2)
N1—C4	1.376 (2)	C13—H13A	0.99
N1—C8	1.447 (2)	C13—H13B	0.99
N1—C7	1.450 (2)	C14—H14A	0.99
C1—C6	1.401 (2)	C14—H14B	0.99
C3—C4	1.406 (2)	C15—C16	1.535 (2)
C3—H3	0.95	C15—C20	1.536 (2)
C4—C5	1.413 (2)	C15—H15	1
C5—C6	1.383 (2)	C16—C17	1.529 (2)
C5—H5	0.95	C16—H16A	0.99
C6—H6	0.95	C16—H16B	0.99
C7—H7A	0.98	C17—C18	1.524 (2)
C7—H7B	0.98	C17—H17A	0.99
C7—H7C	0.98	C17—H17B	0.99
C8—H8A	0.98	C18—C19	1.524 (2)
C8—H8B	0.98	C18—H18A	0.99
C8—H8C	0.98	C18—H18B	0.99
C9—C10	1.534 (2)	C19—C20	1.531 (2)
C9—C14	1.536 (2)	C19—H19A	0.99
C9—H9	1	C19—H19B	0.99
C10—C11	1.532 (2)	C20—H20A	0.99
C10—H10A	0.99	C20—H20B	0.99
C10—H10B	0.99		
			
C1—P1—C15	107.10 (7)	H11A—C11—H11B	107.9
C1—P1—C9	106.66 (7)	C11—C12—C13	111.49 (13)
C15—P1—C9	107.20 (7)	C11—C12—H12A	109.3
C1—P1—Se1	113.31 (5)	C13—C12—H12A	109.3
C15—P1—Se1	111.45 (5)	C11—C12—H12B	109.3
C9—P1—Se1	110.79 (5)	C13—C12—H12B	109.3
C3—C2—C1	121.75 (15)	H12A—C12—H12B	108
C3—C2—H2	119.1	C12—C13—C14	110.87 (13)
C1—C2—H2	119.1	C12—C13—H13A	109.5
C4—N1—C8	120.06 (15)	C14—C13—H13A	109.5
C4—N1—C7	120.11 (14)	C12—C13—H13B	109.5
C8—N1—C7	117.82 (14)	C14—C13—H13B	109.5
C2—C1—C6	117.66 (13)	H13A—C13—H13B	108.1
C2—C1—P1	120.06 (12)	C13—C14—C9	110.75 (13)
C6—C1—P1	122.26 (11)	C13—C14—H14A	109.5
C2—C3—C4	120.95 (14)	C9—C14—H14A	109.5
C2—C3—H3	119.5	C13—C14—H14B	109.5
C4—C3—H3	119.5	C9—C14—H14B	109.5
N1—C4—C3	121.62 (15)	H14A—C14—H14B	108.1
N1—C4—C5	121.10 (15)	C16—C15—C20	111.07 (12)
C3—C4—C5	117.27 (14)	C16—C15—P1	111.22 (10)
C6—C5—C4	121.14 (15)	C20—C15—P1	110.56 (10)
C6—C5—H5	119.4	C16—C15—H15	107.9
C4—C5—H5	119.4	C20—C15—H15	107.9
C5—C6—C1	121.22 (14)	P1—C15—H15	107.9
C5—C6—H6	119.4	C17—C16—C15	111.31 (12)
C1—C6—H6	119.4	C17—C16—H16A	109.4
N1—C7—H7A	109.5	C15—C16—H16A	109.4
N1—C7—H7B	109.5	C17—C16—H16B	109.4
H7A—C7—H7B	109.5	C15—C16—H16B	109.4
N1—C7—H7C	109.5	H16A—C16—H16B	108
H7A—C7—H7C	109.5	C18—C17—C16	111.28 (13)
H7B—C7—H7C	109.5	C18—C17—H17A	109.4
N1—C8—H8A	109.5	C16—C17—H17A	109.4
N1—C8—H8B	109.5	C18—C17—H17B	109.4
H8A—C8—H8B	109.5	C16—C17—H17B	109.4
N1—C8—H8C	109.5	H17A—C17—H17B	108
H8A—C8—H8C	109.5	C19—C18—C17	110.77 (13)
H8B—C8—H8C	109.5	C19—C18—H18A	109.5
C10—C9—C14	110.55 (12)	C17—C18—H18A	109.5
C10—C9—P1	114.22 (10)	C19—C18—H18B	109.5
C14—C9—P1	110.38 (10)	C17—C18—H18B	109.5
C10—C9—H9	107.1	H18A—C18—H18B	108.1
C14—C9—H9	107.1	C18—C19—C20	110.82 (13)
P1—C9—H9	107.1	C18—C19—H19A	109.5
C11—C10—C9	110.26 (13)	C20—C19—H19A	109.5
C11—C10—H10A	109.6	C18—C19—H19B	109.5
C9—C10—H10A	109.6	C20—C19—H19B	109.5
C11—C10—H10B	109.6	H19A—C19—H19B	108.1
C9—C10—H10B	109.6	C19—C20—C15	111.66 (12)
H10A—C10—H10B	108.1	C19—C20—H20A	109.3
C12—C11—C10	111.95 (14)	C15—C20—H20A	109.3
C12—C11—H11A	109.2	C19—C20—H20B	109.3
C10—C11—H11A	109.2	C15—C20—H20B	109.3
C12—C11—H11B	109.2	H20A—C20—H20B	108
C10—C11—H11B	109.2		
			
C3—C2—C1—C6	0.5 (2)	C15—P1—C9—C14	−179.60 (10)
C3—C2—C1—P1	−177.56 (12)	Se1—P1—C9—C14	58.59 (11)
C15—P1—C1—C2	−147.03 (12)	C14—C9—C10—C11	−56.78 (17)
C9—P1—C1—C2	98.45 (13)	P1—C9—C10—C11	178.02 (11)
Se1—P1—C1—C2	−23.72 (14)	C9—C10—C11—C12	55.47 (18)
C15—P1—C1—C6	34.97 (14)	C10—C11—C12—C13	−54.77 (19)
C9—P1—C1—C6	−79.55 (14)	C11—C12—C13—C14	54.96 (18)
Se1—P1—C1—C6	158.28 (11)	C12—C13—C14—C9	−56.65 (17)
C1—C2—C3—C4	−0.8 (2)	C10—C9—C14—C13	57.86 (16)
C8—N1—C4—C3	−2.9 (2)	P1—C9—C14—C13	−174.79 (10)
C7—N1—C4—C3	−166.45 (16)	C1—P1—C15—C16	−172.11 (10)
C8—N1—C4—C5	177.77 (15)	C9—P1—C15—C16	−57.95 (12)
C7—N1—C4—C5	14.2 (2)	Se1—P1—C15—C16	63.44 (11)
C2—C3—C4—N1	−178.82 (15)	C1—P1—C15—C20	64.01 (11)
C2—C3—C4—C5	0.5 (2)	C9—P1—C15—C20	178.16 (10)
N1—C4—C5—C6	179.44 (15)	Se1—P1—C15—C20	−60.45 (11)
C3—C4—C5—C6	0.1 (2)	C20—C15—C16—C17	−53.87 (17)
C4—C5—C6—C1	−0.4 (2)	P1—C15—C16—C17	−177.46 (11)
C2—C1—C6—C5	0.1 (2)	C15—C16—C17—C18	55.73 (18)
P1—C1—C6—C5	178.14 (11)	C16—C17—C18—C19	−57.24 (18)
C1—P1—C9—C10	60.15 (12)	C17—C18—C19—C20	57.01 (19)
C15—P1—C9—C10	−54.31 (12)	C18—C19—C20—C15	−55.75 (18)
Se1—P1—C9—C10	−176.11 (9)	C16—C15—C20—C19	54.09 (16)
C1—P1—C9—C14	−65.15 (12)	P1—C15—C20—C19	178.06 (11)

### Hydrogen-bond geometry (Å, °)

**Table:**

*D*—H···*A*	*D*—H	H···*A*	*D*···*A*	*D*—H···*A*
C15—H15···Se1^i^	1	2.71	3.6546 (15)	157.
C19—H19A···Se1^i^	0.99	3.04	3.8836 (18)	143.

Symmetry codes: (i) *x*, *y*+1, *z*.

## References

[bb1] Altomare, A., Burla, M. C., Camalli, M., Cascarano, G. L., Giacovazzo, C., Guagliardi, A., Moliterni, A. G. G., Polidori, G. & Spagna, R. (1999). *J. Appl. Cryst.* **32**, 115–119.

[bb2] Brandenburg, K. & Putz, H. (2005). *DIAMOND* Crystal Impact GbR, Bonn, Germany.

[bb3] Bruker (2008). *SADABS*, *SAINT* and *XPREP* BrukerAXS Inc., Madison, Wisconsin, USA.

[bb4] Bruker (2011). *APEX2* Bruker AXS Inc., Madison, Wisconsin, USA.

[bb5] Bunten, K. A., Chen, L., Fernandez, A. L. & Poë, A. J. (2002). *Coord. Chem. Rev.* **233–234**, 41–51.

[bb6] Farrugia, L. J. (1999). *J. Appl. Cryst.* **32**, 837–838.

[bb7] Muller, A., Meijboom, R. & Roodt, A. (2006). *J. Organomet. Chem.* **691**, 5794–5801.

[bb8] Muller, A., Otto, S. & Roodt, A. (2008). *Dalton Trans.* pp. 650–657. 10.1039/b712782k 18217121

[bb9] Otto, S. (2001). *Acta Cryst.* C**57**, 793–795. 10.1107/s0108270101006035 11443242

[bb10] Roodt, A., Otto, S. & Steyl, G. (2003). *Coord. Chem. Rev.* **245**, 121–137.

[bb11] Sheldrick, G. M. (2008). *Acta Cryst.* A**64**, 112–122. 10.1107/S0108767307043930 18156677

[bb12] Tolman, C. A. (1977). *Chem. Rev.* **77**, 313–348.

